# Production of Fungal Amylases Using Cheap, Readily Available Agriresidues, for Potential Application in Textile Industry

**DOI:** 10.1155/2014/215748

**Published:** 2014-01-09

**Authors:** Shalini Singh, Sanamdeep Singh, Vrinda Bali, Lovleen Sharma, Jyoti Mangla

**Affiliations:** Department of Biotechnology and Biosciences, Lovely Professional University, Punjab, India

## Abstract

The study aimed at isolation and screening of fungal amylase producer, optimization of solid state fermentation conditions for maximum amylase production by the best amylase producer, and characterization of the crude amylases, so produced. *Aspergillus fumigatus* NTCC1222 showed the highest amylase activity (164.1 U/mL) in secondary screening under SSF conditions and was selected for further studies. The test strain showed maximum amylase production (341.7 U/mL) and supernatant protein concentration (9.7 mg/mL) for incubation period (6 days), temperature (35°C), initial pH (6.0), nutrient salt solution as moistening agent, and beef extract as nitrogen source. Pomegranate peel produced maximum amylase activity, but wheat bran (only slightly lesser amylase activity as compared to that of pomegranate peel) was chosen for further studies, keeping in mind the seasonal availability of pomegranate peel. TLC confirmed the amylase produced to be **α**-type and 60 kDa was the molecular weight of the partially purified amylase. The enzyme showed maximum enzyme activity at pH 6.0, temperature of 55°C, and incubation time of 60 minutes. UV (616.0 U/mL) and chemical (814.2 U/mL) mutation enhanced amylase activity as compared to wild test strain. The study indicates that *Aspergillus fumigatus* NTCC1222 can be an important source of amylase and the crude enzyme, hence obtained, can be cost effectively applied in multiple sections of textile wet processing.

## 1. Introduction

The chemical technology, in particular, has received the great attention due to its hazardous by-products contaminating and polluting the environment and also the high cost of energy inputs for large-scale product manufacture. Search for environmentally compatible and less energy intensive processes has made the biotechnological approach meaningful in the present context. In recent years, the potential of using microorganisms as sources of industrially relevant enzymes has stimulated our interest [1–3], where these enzymes can be used in a large number of industries like food, feed, leather, textile, and paper [4–7] industries. Starch degrading enzymes like amylase have received a great deal of attention because of their technological significance and economic benefits. They are among the most important enzymes and are of great significance in many areas. There are various types of amylases, namely *α*, *β*, and glucoamylases. *α*-amylases (endo-1,4-*α*-D-glucan glucohydrolase, EC 3.2.1.1) are extra cellular enzymes that randomly cleave the 1,4-*α*-D-glycosidic bonds between adjacent glucose units in the linear amylase chain and are classified according to their action and properties. *β*-amylases (*β*-1,4-glucan maltohydrolase, EC 3.2.1.2) are usually of plant origin, but a few microbial strains are also known to produce them. It is an exoacting enzyme that cleaves nonreducing ends of amylose, amylopectin, and glycogen molecule. Glucoamylase (amyloglucosidase, glucanogenic enzymes, starch glucogenase, and exo-1,4-*α*-D-glucan glucanohydrolase (EC 3.1.2.3)) hydrolyses single glucose units from the nonreducing ends of amylose and amylopectin in stepwise manner. Amylases can be derived from various sources, such as plants, animals, and microorganisms, but microbial sources generally meet industrial demands [8] as there is a possibility of increasing the levels of microbial enzyme synthesized by classical genetic techniques, continuous culture selection, induction, or optimization of growth conditions for the enzyme of interest [9–12]. The fungal amylases are preferred over other microbial sources because of their more acceptable GRAS (generally regarded as safe) status, the hyphal mode of growth, and good tolerance to low water activity (*a*_*w*_), and high osmotic pressure conditions make fungi most efficient for bioconversion of solid substrates [13] and thus attracting increasing attention as source of amylolytic enzymes suitable for industrial applications [14–16].

Although most researchers have used liquid culture, which allows greater control of culture conditions such as temperature and pH, SSF has gained huger interest in recent years due to advantages like high yield and high specificity, simple technique, and low moisture contents, which prevent bacterial contamination, capital investment, lower levels of catabolite repression, and better product recovery [13, 17]. Moreover in SSF crude-fermented products can be directly used as enzyme source [18]. Cost of substrates on which enzyme-producing microbes can be cultivated has always been an important factor in production. Complex lignocelluloytic residues are generally considered the best substrates for the SSF processes [19–21]. The nature of the moistening agent and the ratio of substrate to the moistening agent are crucial factors in enzyme production [17]. With the advent of new frontiers in biotechnology, the spectrum of amylase application has widened in many other fields, such as clinical, medicinal [22], and analytical chemistry as well as their widespread application in starch saccharification, food and brewing industries, baking, and preparation of digestive aids. Amylases have also shown to play significant role in textile and detergent industries [23–28]. The natural capability of microorganisms to produce amylases has also been successfully improved using mutational treatment, where both UV and chemical mutagenesis has shown to enhance amylase production. Mutation can further improve amylase production by microorganisms, where UV and chemical treatment [29, 30]. Keeping in view the importance of cheap and efficient production of amylases for industrial processes and the fact that the potential of amylases in textile industry needs to be extensively explored, the present study was carried out to screen out indigenously isolated fungal strains for amylase production for possible application in textile industry.

## 2. Materials and Methods

### 2.1. Pretreatment of Substrates

Lignocellulosic substrates (wheat bran, wheat straw, rice straw, sugarcane bagasse, pomegranate peel, banana peel, pineapple peel, and rye grains) were collected from the local market of Jalandhar, Punjab, India. They were first washed with cold water and subsequently with warm water to remove dirt and impurities. The washed substrates were then dried in sunlight. The dried substrates were grinded in a laboratory grinder and a particle size of 0.5 mm was selected for further studies.

### 2.2. Microorganism

Fungal strains were isolated from soil samples from collected different sites of Jalandhar, Punjab, India, by sweeping off the debris from the top of the soil and scooping the sample of soil (about 100 grams) into a “Ziploc” bag. The soil samples were serially diluted, spread plated on starch agar (SAM) medium (fortified with 0.1 mg/mL streptomycin), and incubated at 25°C. After 3 days of incubation, the plates were stained with Lugol's iodine solution (1% iodine; 2% potassium iodide w/v) and observed for amylase production, as indicated by the appearance of zone of clearance against dark (bluish-black) background.

Stock cultures of purified isolates were maintained on PDA slants, incubated at 25°C for 24 hours, and subsequently stored at 4°C. The cultures were maintained as a suspension of spores and hyphal fragments in 15% (v/v) sterile glycerol at −20°C for long-term preservation.

Amylase positive fungal strains obtained, as detected after amylase plate assay, were further assayed to quantitatively determine actual amylase activities under solid state fermentation (SSF) conditions using wheat bran [31] as the solid substrate. 5 grams of the solid substrate was taken into Erlenmeyer flasks, to which 15 mL of nutrient salt solution (NSS) (in g/L) (potassium dihydrogen orthophosphate 5, ammonium nitrate 5, sodium chloride 1, and magnesium sulphate) was added to adjust moisture levels, thereby maintaining a substrate to moistening agent ratio of 1 : 3 [32]. These flasks were autoclaved at 121°C for 20 minutes at 15 psi pressure. The flasks were cooled, inoculated with two discs of fungal isolates (each of a diameter of 5 mm), and subsequently incubated at 25°C. After 3 days, the contents of flasks were crushed with glass rod and 15 mL of distilled water was added. The contents were then mixed by shaking for 10 minutes at 55°C on a rotary shaker at 200 rpm (rotations per minute). The slurry obtained was squeezed through four layers of cheese cloth. The extract was filtered with a Whatman filter paper 1 and then centrifuged at 5000 rpm for 10 minutes [19, 33]. The filtrate obtained was treated as crude enzyme and the amylase activity was determined by DNS method [34] at 540 nm and reported as U/mL using glucose as a standard. One unit of amylase is defined as the amount of enzyme which releases 1 *μ* mole of reducing sugar per minute (U/mL), with glucose as standard, under the assay conditions described above. The supernatant protein concentration was also determined for the crude enzyme [35] at 660 nm using bovine serum albumin (BSA) as a standard and reported as mg/mL.

The best fungal amylase producer was identified at Plant Pathology Laboratory, Forest Research Institute, Dehradun, India, on the basis of morphological and microscopic features and deposited at National Type Culture Collection (NTCC), Forest Research Institute, Dehradun, India.

Starch hydrolysates were analyzed by thin layer chromatography (TLC) using silica gel plates, where sugar spots of hydrolysates were identified by comparing their Rf values with those similarly obtained for sugar spots of glucose and maltose as reference standards [36].

### 2.3. Optimization of Production of Amylase under SSF

The effect of incubation period on amylase activity was determined by incubating the inoculated flasks for variable incubation period (1 to 10 days) at pH 6.0 using wheat bran as carbon source and beef extract as the nitrogen source, at a temperature of 37°C. The wheat bran was moistened with NSS in the ratio of 1 : 3 as previously described. The enzyme was harvested after every 24 hours up to 10 days on incubation and amylase activity [34] and supernatant protein concentration [35] were determined as described previously. Similarly, the inoculated flasks were incubated at variable temperatures (25°C, 30°C, 35°C, 40°C, 45°C, and 50°C) using wheat bran as carbon source at pH of 6.0 for optimized incubation period and variable initial pH (3, 3.5, 4, 4.5, 5, 5.5, 6, 6.5, 7, 7.5, and 8) using wheat bran as carbon source for optimized incubation time and temperature. The pH of the fermentation medium was not monitored after autoclaving and only the initial pH was maintained. The enzyme was subsequently harvested and amylase activity [34] and supernatant protein concentration [35] were determined as previously described.

A total of 19 different substrates (9 lignocellulosic substrates, singly and in combinations) were tested to see their effect on amylase production. The flasks were prepared, inoculated, and incubated as above and the amylase activity [34] and supernatant protein concentration were determined [35].

The effect of nitrogen source (peptone, beef extract, tryptone, yeast extract, and ammonium nitrate as nitrogen sources) and moistening agents, namely NSS, distilled water, and tap water [21, 37] on amylase activity was also determined under the above-optimized conditions of incubation period, temperature, initial pH, and substrate [38]. The amylase activity and supernatant protein concentration were determined as described earlier.

### 2.4. Production and Partial Characterization of Crude Amylase by Test Fungi

The test fungal strain was subjected to optimized conditions of incubation temperature (37°C), incubation period (6 days), and initial pH (6.0), using NSS as the moistening agent, solid to liquid ratio (1 : 3), wheat bran as carbon source, and yeast extract as the nitrogen source under SSF. The amylase activity and supernatant protein concentration were then determined. The crude amylase preparation obtained from test fungi under optimized conditions of SSF was further tested for the array of enzymes present in the crude enzyme preparation that were produced under the above-mentioned optimized conditions. Filter paper assay (FPase assay) was carried [39] using filter paper strips (6 cm × 1 cm) as the substrate and spectrophotometrically determining the reducing sugars [34] produced at a wavelength of 550 nm. The enzyme activity was expressed in terms of filter paper unit (FPU) per mL of undiluted culture filtrate. A filter paper unit (FPU) is defined as *μ*g of reducing sugar liberated in one hour under standard assay conditions. Carboxymethyl cellulase (CMCase) activity was determined [39] using carboxymethyl cellulose (CMC) as the substrate and expressed as international unit (IU), defined as the amount of enzyme that produces one *μ* mole of glucose released per minute [34]. Pectinase activity was assayed by the colorimetric method at 595 nm [34] using pectin and glucose as the standard. One unit of pectinase activity was defined as the amount of enzyme which liberated 1 *μ*m glucose per min. Xylanase activity was determined [34] at 540 nm, using birchwood xylan as the substrate. The amount of reducing sugar liberated was quantified using xylose as standard. One unit of xylanase is defined as the amount of enzyme that liberates 1 *μ*mol of xylose equivalents per minute under the assay conditions. Laccase activity was determined as per the method of Ride [40].

The crude enzyme was subjected to variable pH (3.0, 3.5, 4.0, 4.5, 5.0, 5.5, 6.0, 6.5, 7.0, 7.5, and 8.0), incubation temperature (35°C, 45°C, 55°C, 65°C, and 75°C), and incubation time (30, 60, 90, and 120 minutes), and the amylase activity was determined [34].

The molecular weight of alpha-amylase of test fungi was determined by subjecting the enzyme preparation to ammonium sulphate (0–40% and 40–70%) [41] precipitated and subsequently dialysed (50 kDa) [41] enzyme to SDS-PAGE analysis as per the method described by Laemmli [42] for the determination of molecular weight. For SDS-PAGE, medium range protein marker (3–205 KDa) was used. For zymogram analysis, after electrophoresis the gel was washed twice with 0.5% (v/v) triton X-100 for 15 min to remove SDS. The gel was incubated in 2.0% (w/v) soluble starch in buffer at 55°C for 60 min. The gel was then kept in the same buffer for 10 min at 55°C and then stained with iodine solution (0.3% I_2_ in 3% KI) for 10 min.

Kinetic studies were carried out for the said enzyme using different starch (5.0–75 mg/mL) concentrations for the determination of *K*_*m*_ and *V*_max_ using lineweaver burk plot by estimation of amylase activity [34].

### 2.5. Effect of Mutation on Amylase Production by the Test Fungi

Plate cultures were exposed to UV radiations (254–260 nm) using UV lamp (1.6 × 102 J/m^2^/S energy) for varying time intervals (3, 5, 10, 15, and 20 mins) and kept in the dark for the stabilization of thymine-thymine dimers [43]. The amylase activity [34] of stably mutated test strain was determined as previously described. The test fungus was also subjected to chemical mutation using EMS-EtBr mutation [44–46] and the stably mutated strains were evaluated for amylase activity.

### 2.6. Statistical Analysis

All the cultures were replicated in triplicates and the results are mean standard deviation (±SD) of the value.

## 3. Results and Discussion

### 3.1. Microorganism

A total of 7 fungal strains were found to be positive for amylase production, as determined by measuring the width of the clear zone (zone of hydrolysis) formed around the fungal colonies (Table 1). The fungal strains exhibiting a zone of hydrolysis of more than 0.5 cm were selected for further studies. Thus, S1, S2, S3, and S4 (the amylase positive fungal strains were temporarily named arbitrarily as S1, S2, etc., where the numerics indicate the site of sample collection) were further subjected to secondary screening to determine their actual amylase activity under conditions of solid state fermentation (SSF), as amylase plate assay indicated that fungal strains can be arranged in the following descending order in terms of diameter of zone of hydrolysis: S3 (4.0 cm) > S4 (3.6 cm) > S2 (2.6 cm) > S1 (1.5 cm). The secondary screening (Table 2) showed similar results, wherein S3 (Table 3) exhibited the highest amylase activity (164.1 U/mL) under SSF using wheat bran as the solid substrate after 3 days of incubation period. Similarly, S2, S4, and S1 showed decreased levels of amylase activity in the following descending order of decreasing enzyme activity: S2 (143.7 U/mL) > S4 (113.4 U/mL) > S1 (105.1 U/mL). Thus, fungal isolate S3 was selected for further studies and it was identified as *Aspergillus fumigatus* NTCC 1222 at Laboratory of Plant Pathology, Forest Research Institute, Dehradun, India, on the basis of morphological features and microscopic examination. Glucose and maltose were found to be the main products on subjecting the crude amylase of the test strain to TLC analysis, confirming that the amylase produced by the above organisms was of *α*-type.

### 3.2. Optimization of Production of Amylase under SSF

As reported in Table 3, amylase production (262.3 U/mL) and the corresponding supernatant protein concentration (7.1 mg/mL) increased with an increase in the incubation time, reaching the maximum on the 6th day of incubation period. After 6 days of incubation, amylase production decreased to 221.5 U/mL and 193.9 U/mL on the 7th and 8th day of incubation period, respectively. The observed decrease is most likely due to substrate inhibition [47].  Table 4 shows amylase production and supernatant protein concentration at different temperatures varying from 25°C to 50°C. The maximum amylase production (337.4 U/mL) and supernatant protein concentration (7.9 mg/mL) were observed at 35°C when the microbial culture was subjected to amylase production at different temperatures varying from 25°C to 50°C. Below 35°C, amylase production decreased by 13.70% at 30°C. At 40°C, the amylase activity and supernatant protein concentration decreased by 10.50% and 10.10%, respectively, indicating mesophilic nature of the test fungal strain. The amylase production and supernatant protein concentration (Table 5) were the maximum (339.1 U/mL, 8.1 mg/mL, resp.) at an initial pH 6.0. At pH below the optimum, a decrease of 17.80% and 7.10% (pH 4.0 and 5.0 resp.) was observed in amylase activity. Similarly, above the optimum pH, the amylase activity decreased by 14.77% and 32.85% (pH 7.0 and 8.0 resp.). The results indicated that slightly acidic pH was required for the amylase to be produced by test strain under SSF. As Table 6 shows, the maximum amylase activity was observed for pomegranate peel (335.4 U/mL) as the solid substrate, but owing to its seasonal availability (increased cost), the next best performer, that is, wheat bran (327.2 U/mL), which showed only a very slight decrease in amylase activity as compared to pomegranate peel as the substrate, was selected for further studies owing to the fact that wheat bran is cheaply and readily available and it contains sufficient nutrients that support good microbial growth and high yield of enzymes as well [47, 48]. The rest of the substrates showed the following increasing order of enzyme activity: RS (131.1 U/mL) > RS + WS (131.2 U/mL) > WB + RS (153.3 U/mL) > Rye grains (163.1 U/mL) > WS (182.1 U/mL) > BAN + RS (207.2 U/mL) > WB + WS (212.2 U/mL) > BAN + SB (215.2 U/mL) > WS + SB (241.3 U/mL) > WB + BAN (277.5 U/mL) > BAN (289.3 U/mL) > PG + PA (292.1 U/mL) > BAN + PA (299.2 U/mL) > PA (321.2 U/mL) > SB (324.3 U/mL) > PG + WS (325.2 U/mL) > PG (335.4 U/mL). Wheat bran as the best substrate for amylase production has also been reported by other researchers [48, 49].  Table 7 indicates that beef extract served as the best nitrogen source for amylase production (341.6 U/mL) and supernatant protein concentration (9.3 mg/mL), followed by ammonium nitrate (327.9 U/mL) > peptone (299.1 U/mL) > yeast extract (277.5 U/mL) > tryptone (258.5 U/mL), as nitrogen source in particular decreasing values for amylase production and supernatant protein concentration. Vardhini et al. [50] have also reported beef extract as the best nitrogen source for amylase production by *Aspergillus* sp. [51]. The maximum amylase production (342.8 U/mL) and supernatant protein concentration (9.6 mg/mL) were observed with NSS as the moistening agent (Table 8). A decrease of 13.53% with tap water and 2.59% with distilled water for amylase activity, compared to NSS, was observed. The influence of moistening agent on microbial growth and product biosynthesis might be attributed to its impact on the physical properties of solid substrate [17]. The results also confirm the positive influence of nutrients present in NSS for production of amylase by the test fungi.

### 3.3. Production and Partial Characterization of Crude Amylase by Test Fungi

As shown in Table 9, under optimized conditions of SSF (wheat bran as a solid substrate, NSS as moistening agent, beef extract as nitrogen source, incubation period of 6 days at 35°C, and pH 6.0), amylase activity and supernatant protein concentration were found to be 341.7 U/mL and 9.7 mg/mL, respectively. The crude enzyme preparation obtained from test fungi was characterized by the array of enzymes that were produced under the optimized conditions of amylase production by SSF. The idea was to evaluate the enzyme preparation for subsequent use in multiple sections of textile industry so as to decrease the overall cost of textile processing. The total cellulase, CMCase, pectinase, and laccase activities were found to be 0.033 IU/mL, 0.046 IU/mL, 0.587 IU/mL, and 106 U/mL, respectively.

Further, the effect of incubation temperature, pH, and incubation time was also studied. The maximum amylase activity (340.1 U/mL) was obtained at pH 6.0 (Table 10) when crude amylase preparation was incubated at different pH for 30 minutes at 40°C. Further increase in pH leads to decrease of 2.52% (pH 7.0) and 9.90% (pH 8.0) in amylase activity. At pH 4.0 and 3.0, the enzyme activity decreased by 8.64% and 8.58%, respectively, as compared to the maximum amylase activity. Similarly, the maximum amylase activity (346.6 U/mL) was obtained at 55°C (Table 11) when crude amylase preparation incubated at different temperature for 30 minutes at optimized pH. Decrease in temperature below the optimum led to decrease of 7.96% (45°C) and 19.04% (35°C) and increase in temperature above optimum led to decrease of 9.95% (65°C) and 45.06% (75°C) in amylase activity. The results indicate thermotolerant nature of the said crude amylase preparation which promises potential compatibility of the said enzyme with conventional industrial chemical processes. The maximum amylase activity (356.2 U/mL) was obtained at an incubation period of 60 minutes (Table 12) when crude amylase preparation incubated at optimized temperature and pH.

The specific activity of the enzyme was 44.2 U/mg for crude enzyme, 309.3 U/mg after ammonium sulphate precipitation and 893.7 U/mg after dialysis. Thus, 7- and 20-fold purification was obtained by ammonium sulphate precipitation and dialysis, respectively. The molecular weight of amylase produced by the test strain was found to be 60 kDA by SDS-PAGE analysis. The amylase activity was confirmed by zymogram analysis as a pale yellow band in dark-colored gels [52].

**Figure 1:**
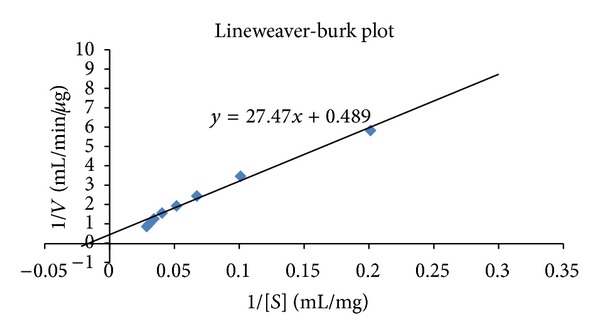
Lineweaver-Burk plot for determining *K*_*m*_ and *V*_max_.

The Michaelis-Menten constant (*K*_*m*_) was determined by Lineweaver-Burk plot using starch as a substrate (Figure 1). The *K*_*m*_ value of *α*-amylase of mutated strain, as calculated from the slope of the graph, was found to be 56.03 mg/mL. The maximum reaction rate (*V*_max_) was found to be 2.04 *μ*g/mL/min.

### 3.4. Effect of Mutation on Amylase Production by the Test Fungi

The exposure of wild strain of *A. fumigatus* NTCC 1222 to UV treatment was optimized (data not given) and it was found that the UV-mutated strain of *A. fumigatus* NTCC 1222 showed an increase of 44.52% in amylase activity over amylase activity for the wild strain of the said fungus. The chemically (EMS-EtBr) mutated strain reported an increase of 58.03% in amylase activity as compared to wild strain of the said fungus. The UV-mutated strain showed an increase of 67.96% for total cellulase activity, an increase of 22.03% for CMCase activity, a decrease of 95.22% for pectinase activity and a decrease of increase of 10.0% for laccase activity, as compared to the respective enzymes obtained from wild strain of *A. fumigatus* NTCC 1222. For chemically-mutated strain, the total cellulase, CMCase, pectinase and laccase activities were respetively increased by 65.97%, decreased by 8.69%, decreased by 80.91%, decreased by 12.00%, as compared to respective enzymes obtained from wild strain of *A. fumigatus* NTCC 1222 (Table 13).

## 4. Conclusion

The present study attempts to explore the potential of indigenously isolated fungal strains to produce amylases using cheap lignocellulosic substrate for their potential application in textile industry. A total of 7 strains were isolated from soil samples collected from different locations of Jalandhar, Punjab, India. Out of a total of 7 fungal strains isolated from soil, 4 were found to be amylase positive and they showed variable amylase activities under conditions of SSF using wheat bran as the cheap lignocellulosic substrate. *Aspergillus fumigatus* NTCC 1222, exhibiting the best amylase activity among the isolate, produced 341.7 U/mL of amylases under optimized conditions of incubation period (6 days), incubation temperature (35°C), and incubation pH (6.0), using nutrient salt solution (NSS) as the moistening agent, beef extract as the nitrogen source, and wheat bran as the solid substrate. The crude enzyme preparation, so obtained from the test strain under optimized conditions of SSF, was found to be thermoalkali stable, as it retained about 90% of its maximum activity at pH 8.0 and more than 50% of its maximum activity at a temperature of 75°C. UV-mutated and chemical-mutated strains were, respectively, found to exhibit an increase of 44.52 and 58.03% in amylase activity as compared to wild strain of *Aspergillus fumigatus* NTCC1222. The results are significant as they indicate that the enzyme will be active even under the presence of high temperatures and pH, thereby favoring its use under harsh conditions of textile wet processing. The crude amylase preparation is also found to contain important industrial accessory enzymes, which add to the value of the said enzyme preparation for industrial applications. Use of a single enzyme preparation in multiple steps of industrial processing has the potential to improve the economics of the overall process. Thus, the future work deals with application of the crude enzyme of *Aspergillus fumigatus *NTCC1222 in multiple sections of textile wet processing so as to explore cost-effective usage of enzymes.

## Figures and Tables

**Table 1:** Primary screening to isolate amylase positive fungal strains.

Strains	Location	Width of clear zones on SAM (cm)
S1	Wheat flour mill 1 (Mohalla Gobind Garh, Jalandhar, Punjab, India)	1.5
S2	Wheat flour mill 2 (Kapurthala Road, Jalandhar, Punjab, India)	2.6
S3	Potato field 1 (Guru Tegh Bahadur Nagar, Jalandhar, Punjab, India)	4.0
S4	Wheat flour mill 3 (Pathankot Bypass, Jalandhar, Punjab, India)	3.6
S5	Potato field 2 (S.K Farms, G.T Road, Jalandhar, Punjab, India)	—
S6	Potato field 3 (Nakodar Road, Jalandhar, Punjab, India)	—
S7	Kitchen garden (Model Town, Jalandhar, Punjab, India)	—

—: no amylase activity.

Growth conditions: incubation time, days: 3; incubation temperature, °C: 25.

**Table 2:** Secondary screening of fungal isolates for amylase activity.

Strains	Amylase activity (U/mL)
S1	105.1 ± 2.1
S2	143.7 ± 1.4
S3	164.1 ± 1.7
S4	113.4 ± 0.9

±: standard deviation from the mean.

Fermentation conditions: incubation period, days = 3; temperature, °C = 25; pH = 6.0.

Assay conditions: incubation time, minutes = 30; temperature, °C = 40; pH = 6.0.

**Table 3:** Effect of incubation period on amylase production and supernatant protein concentration.

Incubation period (days)	Amylase activity (U/mL)	Protein concentration (mg/mL)
1	106.4 ± 2.5	3.3 ± 0.9
2	134.4 ± 1.8	3.9 ± 0.81
3	166.7 ± 1.6	4.0 ± 0.11
4	199.4 ± 0.6	4.6 ± 0.46
5	227.6 ± 1.4	6.1 ± 0.2
6	262.3 ± 1.3	7.1 ± 0.45
7	221.5 ± 0.6	6.3 ± 0.95
8	193.9 ± 2.1	5.4 ± 0.19

±: standard deviation from the mean.

Fermentation conditions: temperature, °C = 25; pH = 6.0.

Assay conditions: incubation time, minutes = 30; temperature, °C = 35; pH = 6.0.

**Table 4:** Effect of temperature on amylase production and supernatant protein concentration.

Temperature (°C)	Amylase activity (U/mL)	Protein concentration (mg/mL)
25	262.8 ± 0.9	7.3 ± 0.42
30	291.1 ± 1.1	7.4 ± 0.5
35	337.4 ± 0.5	7.9 ± 0.14
40	301.8 ± 0.9	7.4 ± 0.23
45	264.0 ± 1.2	7.1 ± 0.33
50	229.5 ± 2.3	6.3 ± 0.21

±: standard deviation from the mean.

Fermentation conditions: incubation period, days = 6; pH = 6.0.

Assay conditions: incubation time, minutes = 30; temperature, °C = 40; pH = 6.0.

**Table 5:** Effect of initial pH on amylase production and supernatant protein concentration.

pH	Amylase activity (U/mL)	Protein concentration (mg/mL)
3.0	217.3 ± 0.3	5.3 ± 1.1
4.0	278.6 ± 1.7	6.2 ± 0.97
5.0	315.0 ± 1.2	7.8 ± 0.54
6.0	339.1 ± 2.4	8.1 ± 0.18
7.0	289.0 ± 2.1	7.8 ± 0.21
8.0	227.7 ± 0.3	6.8 ± 1.25

±: standard deviation from the mean.

Fermentation conditions: incubation period, days = 6; temperature, °C = 35.

Assay conditions: incubation time, minutes = 30; temperature, °C = 40; pH = 6.0.

**Table 6:** Effect of substrates on amylase production and supernatant protein concentration.

Substrate	Amylase activity (U/mL)	Supernatant protein concentration (mg/mL)
Wheat bran (WB)	327 ± 2.7	8.1 ± 0.72
Wheat straw (WS)	182 ± 1.2	6.8 ± 0.10
Rice straw (RS)	131 ± 1.7	4.2 ± 0.07
Sugarcane bagasse (SB)	324 ± 1.8	7.9 ± 0.27
Pomegranate peel (PG)	335 ± 2.1	8.3 ± 0.08
Pineapple peel (PA)	321 ± 1.1	8.1 ± 0.23
Banana peel (BAN)	289 ± 0.9	8.2 ± 0.39
Rye grains (RG)	163 ± 1.3	6.1 ± 0.33
Starch	326 ± 1.3	7.9 ± 0.33
WS + SB (1 : 1)	241 ± 0.9	6.9 ± 0.88
WB + RS (1 : 1)	153 ± 1.4	4.7 ± 1.22
WB + WS (1 : 1)	212 ± 1.6	7.2 ± 0.99
WB + BAN (1 : 1)	277 ± 1.9	7.6 ± 1.10
BAN + PA (1 : 1)	299 ± 1.3	7.3 ± 0.04
BAN + RS (1 : 1)	207 ± 0.7	6.3 ± 0.19
PG + PA (1 : 1)	292 ± 2.5	7.9 ± 0.61
PG + WS (1 : 1)	327 ± 2.1	8.0 ± 0.13
BAN + SB (1 : 1)	215 ± 1.2	8.5 ± 0.08
RS + WS (1 : 1)	131 ± 2.2	4.9 ± 0.31

±: standard deviation from the mean.

Fermentation conditions: incubation period, days = 6; temperature, °C = 35.

Assay conditions: incubation time, minutes = 30; temperature, °C = 40; pH = 6.0.

**Table 7:** Effect of nitrogen sources on amylase production and supernatant protein concentration.

Complex nitrogen source (1% w/v)	Amylase activity (U/mL)	Protein concentration (mg/mL)
Ammonium nitrate	327.9 ± 1.1	8.0 ± 0.13
Peptone	299.1 ± 2.1	7.7 ± 1.09
Beef extract	341.6 ± 1.5	9.3 ± 0.27
Yeast extract	277.5 ± 1.3	6.9 ± 0.42
Tryptone	258.5 ± 0.7	6.4 ± 0.44

±: standard deviation from the mean.

Fermentation conditions: incubation period, days = 6; temperature °C = 35; pH = 6.0.

Assay conditions: incubation time, minutes = 30; temperature °C = 40; pH = 6.0.

**Table 8:** Effect of moistening agents on amylase production and supernatant protein concentration.

Moistening agent	Amylase activity (U/mL)	Protein concentration (mg/mL)
Tap water	296.4 ± 0.2	6.1 ± 0.28
Distilled water	333.9 ± 1.9	8.1 ± 0.13
NSS	342.8 ± 2.6	9.6 ± 0.90

±: standard deviation from the mean.

Fermentation conditions: incubation period, days = 6; temperature, °C = 35; pH = 6.0.

Assay conditions: incubation time, minutes = 30; temperature,°C = 40; pH = 6.0.

**Table 9:** Production of amylase under optimized conditions of SSF.

Amylase activity (U/mL)	FPase activity (IU/mL)	CMCase activity (IU/mL)	Pectinase activity (IU/mL)	Laccase activity (U/mL)	Protein concentration (mg/mL)
341.7 ± 1.6	0.033	0.046	0.587	106	9.7 ± 0.61

±: standard deviation from the mean.

Fermentation conditions: incubation period, days = 6; temperature,°C = 35; pH = 6.0.

Assay conditions: incubation time, minutes = 30; temperature, °C = 40; pH = 6.0.

**Table 10:** Effect of pH on crude amylase preparation.

pH	Amylase activity (U/mL)
3.0	310.9 ± 1.6
4.0	310.7 ± 1.3
5.0	322.9 ± 0.2
6.0	340.1 ± 1.9
7.0	331.5 ± 2.2
8.0	306.4 ± 2.3

±: standard deviation from the mean.

Fermentation conditions: incubation period, days = 6; temperature, °C = 35; pH = 6.

Assay conditions: incubation time, minutes = 30; temperature,°C = 40.

**Table 11:** Effect of temperature on crude enzyme preparation.

Temperature	Enzyme activity (U/mL)
35	338.7 ± 1.6
45	345.3 ± 0.1
55	346.6 ± 1.2
65	321.1 ± 0.8
75	299.4 ± 0.7

±: standard deviation from the mean.

Fermentation conditions: incubation period, days = 6; temperature, °C = 35; pH = 6.

Assay conditions: incubation time, minutes = 30; pH = 5.0.

**Table 12:** Effect of incubation time on crude amylase preparation.

Time (minutes)	Amylase activity (U/mL)
30	344.7 ± 0.9
60	356.2 ± 2.2
90	319.0 ± 1.8
120	273.5 ± 1.1

±: standard deviation from the mean.

Fermentation conditions: incubation period, days = 6; temperature, °C = 35; pH = 6.

Assay optimized conditions: temperature, °C = 55; pH = 5.0.

**Table 13:** Effect of mutation on amylase production by the test fungi.

Enzyme activity (+/−: % difference compared to wild strain)	Microbial strain	
	UV mutated	Chemically mutated
Amylase activity	+44.52	58.03
FPase activity	+67.96	+65.97
CMCase activity	+22.03	−8.69
Pectinase activity	−95.22	−80.91
Laccase activity	−10.00	−12.00
